# Clinical reasoning amongst paramedics using nebulised β₂ agonists to treat acute asthma exacerbations: a qualitative study

**DOI:** 10.1038/s41533-024-00383-w

**Published:** 2024-09-06

**Authors:** Craig Mortimer, Dimitra Nikoletou, Ann Ooms, Julia Williams

**Affiliations:** 1 Research and Development Department, South East Coast Ambulance Service NHS Foundation Trust, Crawley, UK; 2 Faculty of Health, Science, Social Care and Education, Kingston University of London, Kingston upon Thames, London, UK; 3 St George’s School of Health and Medical Sciences, City St George’s, University of London, Tooting, London, UK

**Keywords:** Asthma, Respiratory signs and symptoms

## Abstract

The heterogeneous nature of asthma results in a wide range of presentations during exacerbation. Despite UK pre-hospital management guidelines focusing on β₂ agonists, variables such as cause, severity, underlying health, comorbidities, and drug side effects can often make emergency treatment optimisation difficult. This article examines paramedics’ methods of observing, perceiving, interpreting, and treating asthma with β₂ agonists, often acting on limited information in rapidly evolving situations. We recruited paramedics from a single UK National Health Service ambulance Trust for qualitative semi-structured interviews. Responses underwent framework analysis to identify data similarities and differences. Fifteen qualitative interviews with paramedics revealed three main themes affecting patient management: clinician experience of presentation, adaptation of patient management approaches, and severity of side effects. Paramedics felt their ability to manage various asthma presentations was enhanced through guideline adaptation based on their own clinical experience and understanding of β₂ agonist side effects, allowing tailored responses based on a set of reinforcing factors. Inductive analysis revealed additional complexities within these themes, such as anxiety and diabetes, which may influence β₂ agonist administration and result in multiple care pathways being initiated during exacerbation. Paramedic care mirrors asthma’s complexity, accounting for a range of characteristics. A dynamic, critically thought approach enables patient management to be based on the presenting conditions rather than strict adherence to a single algorithm. Comprehending the complexities and variables in treatment can be crucial to how paramedics rationalise their treatment and optimise the care provided.

## Introduction

Globally the prevalence of asthma continues to increase, resulting in greater demand for prehospital and intrahospital services, with predicted increases in morbidity and mortality^1^. According to the National Review of Asthma Deaths^2^, optimisation of basic primary care can achieve significant reductions in these morbidity and mortality rates. Therefore, it is imperative that first-line treatment in the emergency setting and support during recovery is optimised in terms of effectiveness and timeliness^3,4^.

However, despite asthma’s heterogeneous nature, including different phenotypes and compounding variables such as comorbidities, the standard treatment for patients experiencing an exacerbation of asthma has remained constant for over forty years and involves the use of bronchodilator drugs (β₂ agonists) to relieve the associated bronchospasm^5,6^. Notwithstanding, recent studies suggest that the use of β₂ agonists may result in negative short and long-term effects, with adverse physiological effects, such as arrhythmia, potentially resulting in reciprocal changes including altered blood pressures and modified respiratory patterns, or metabolic changes including increased blood glucose levels, all which can cause greater ventilation/perfusion (V/Q) mismatch^7–12^.

Nonetheless, current international asthma guidelines published by the Global Initiative for Asthma (GINA) continue to state that asthma patients in exacerbation should receive *‘*repetitive administration of short-acting inhaled bronchodilators, early introduction of systemic corticosteroids, and controlled flow oxygen supplementation*’* which can be further supported with additional drugs as required, including ipratropium bromide and magnesium^13^. However, in contrast to these guidelines and those published by the British Thoracic Society^14^, UK ambulance services primarily focus on the use of β₂ agonists without the supplementation of corticosteroids, unless the exacerbation is severe or life-threatening in nature.

Given the divergence of asthma patients, the varying degrees of airway inflammation and the variety of physiological changes induced by first-line treatment during exacerbation, this study sought to explore the perceptions and experiences of ambulance paramedics managing acute exacerbations of asthma in the pre-hospital setting and how they alter their management in response to changes in physiological observations and in consideration of other variables.

This focus is important as healthcare providers may not record these observations or perceive them as problematic in the presence of an asthma exacerbation^8,12,15,16^. Furthermore, patients experiencing an exacerbation may be managed solely by paramedics without subsequent examination by a hospital or General Practitioner (GP)^17,18^.

## Methods

### Study design

**Fig. 1:**
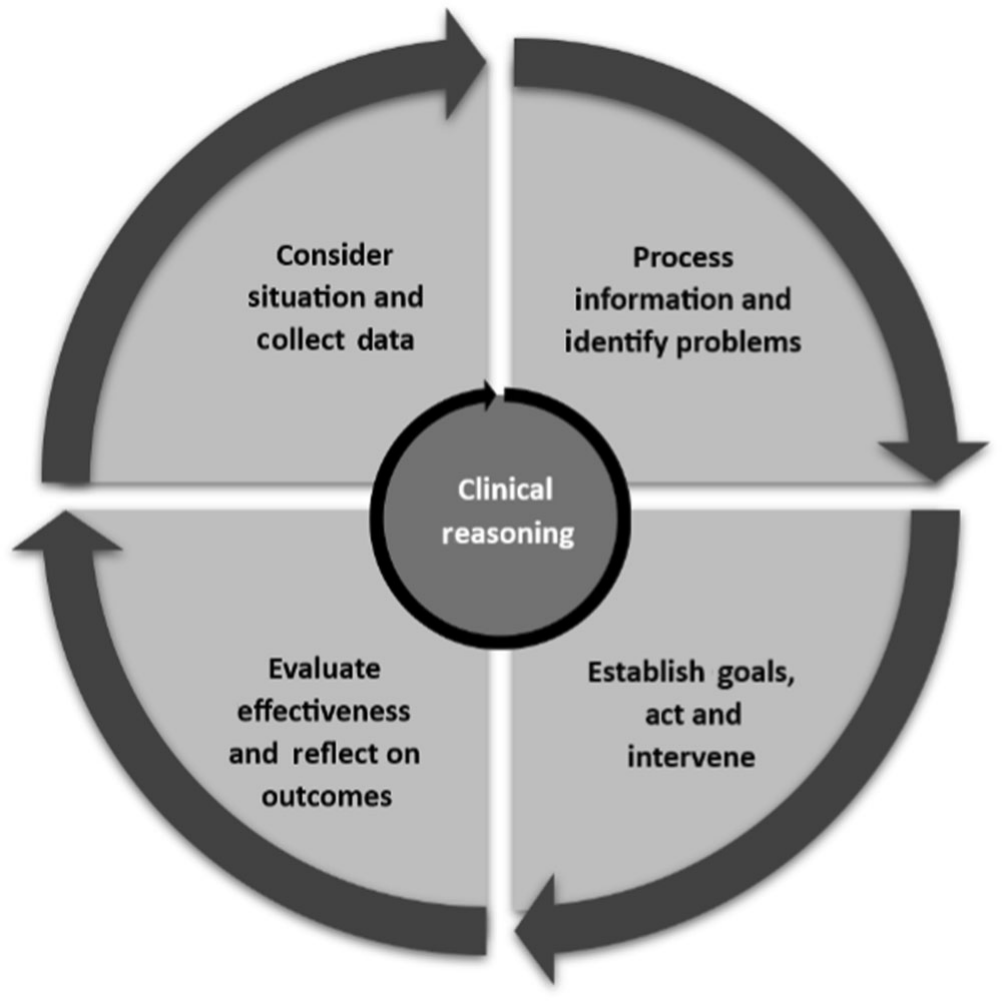
Clinical reasoning cycle. Cyclical approach used by healthcare professionals to make a clinical judgement.

An exploratory study employed semi-structured interviews devised using a schedule (S1 Table) developed applying current literature, the *‘*clinical reasoning cycle*’* framework (Fig. 1)^19^ and based on an interpretivism philosophy with epistemology construct. By referring to the framework during schedule development, it was hoped that all aspects of clinical reasoning could be considered during interviews.

The interview schedule was primarily developed by CM an experienced paramedic with extensive knowledge and experience of asthma in a variety of settings, with the support of two academics (ND, AO) and a paramedic practitioner with expertise in pre-hospital emergency and primary care. The interviews focused on five key priori categories with additional sub-themes (Table 1).

**Table 1:** Interview schedule: categories and questions.

Categories	Indicative questions
Prevalence/background experience	Frequency of attending asthma patients? Levels of severity? General age range?
Standard management	General management process? Main drugs used? Dosage required to control exacerbation. Frequency ipratropium bromide or hydrocortisone administered?
Pharmacodynamics	Experience of salbutamol side effects? Frequency of suggested side effects? Salbutamol discontinued due to side effects. When do side effects become problematic? How is risk of side effects balanced?
Comorbidity and multimorbidity	Frequency of presenting comorbidity? Required change to treatment? Unknown comorbidity presenting?

Health and Care Professions Council registered paramedics with experience in face-to-face management of at least three acute asthma exacerbations during the preceding 12-month time frame, were recruited from a single UK ambulance Trust using Trust-wide adverts between 1st April 2021 and 15th December 2021.

During the timeframe, 15 paramedics volunteered to participate in online interviews. A view taken by Guest et al. ^20^ and Vasileiou et al. ^21^ amongst others, suggest that data saturation, especially when considering data quality and commonality, is key to effective qualitative research. Given the study location and target population, it was expected that data saturation would be reached with this number of participants. The sample outline for participants is shown in Table 2. A description of the specified roles can be found in the S2 Table. Lasting 45 min on average and using Microsoft Teams, interviews were digitally recorded and transcribed verbatim.

**Table 2:** Interview sample outline.

Clinical qualification	Participant numbers	Sex
Experienced Paramedics	11	Male × 8 Female x 3
Newly qualified paramedics	2	Male × 1 Female × 1
Paramedic practitioners	2	Male × 1 Female × 1

### Data analysis

Framework analysis^22,23^ was used to analyse the interviews, allowing a descriptive paradigm to facilitate a deductive and inductive approach. The initial deductive approach used the Joint Royal Colleges Ambulance Liaison Committee (JRCALC) guidelines to develop priori coding. Developed by the committee through consultation with representatives from a wide range of healthcare experts, including paramedics, nurses and GPs, these central guidelines are used by all ambulance clinicians within National Health Service (NHS) ambulance services, and facilitated data coding of collected data into sub-themes aligned to existing literature relevant to the administration of β₂ agonists. Further analysis allowed these codes to be grouped and combined to provide targeted core themes that influence how paramedics manage asthma patients. Inductive analysis was used where appropriate to identify the complexities faced by paramedics when managing patients with asthma.

All data were reviewed by CM and DN for accuracy and consensus regarding coding^24^.

### Ethical and regulatory considerations

This article observes the Standards for Reporting Qualitative Research^25^ and the study was approved by the Health Research Authority (IRAS 291421). Further ethical approval (KU2756) was obtained from Kingston University, London. Local research governance was gained from the participating Trust and prior to the interview participants provided informed consent both verbally and in writing having reviewed a Participant Information Sheet.

### Reporting summary

Further information on research design is available in the Nature Research Reporting Summary linked to this article.

## Results

### Clinical reasoning themes

Participants’ responses provided a range of codes across the priori categories (Table 1). When combined and analysed for commonality the deductively analysed data provided three core themes that focused on the participants’ use of clinical reasoning:

- Clinician experience of presentation
- Adaptation of patient management approaches
- Severity of side effects

Inductive analysis provided fewer codes that whilst associated with the priori category of comorbidity/multimorbidity, referenced variables outside of those stated within the JRCALC’s asthma guideline. These variables are noted as appropriate to support the following themes.

### Clinician experience of presentation

On average participants encountered a patient with asthma every month, with the majority of calls being for patients experiencing mild-moderate exacerbations, rather than severe or life-threatening.

*‘Most of the ones who we go to at the moment are quite mild. Actually. I haven’t been to many severe asthma patients in quite some time.’ (32110)*

*‘I would say the majority are mild to moderate and sometimes edging to severe. Life threatening in my experience is rare.’ (32212)*

No consensus could be reached on the average duration of an exacerbation. On average nine of the fifteen participants had experienced exacerbations resolving within 30 min. However, some noted that the duration is not always clear, as once patients are handed over to hospital staff, paramedics tend to move on to the next call. Participants found that mild exacerbations can resolve in the patient’s home environment if the patient’s personal inhaler takes effect before paramedics arrive and/or before the ambulance arrives and provides reassurance.

*‘Sometimes they’ve called us out and their pumps have controlled it mostly before us arriving. So, we put them on a single salbutamol neb. and it controls that in the five minutes it takes to run through.’ (4729)*

*‘The problem is we may have a patient for forty minutes and then hand them over to hospital staff and they are still struggling to breath, so the final time is unknown.’ (1646)*

*‘I think there’s probably a degree of anxiety around not being able to breath that comes with a worsening asthma attack, and I think that although the salbutamol takes effect, it’s getting that person to calm down and the body just to relax back to normal.’ (1626)*

All participants agreed that the patient’s general health status, underlying medical condition, environment, and initial trigger are important for the effective management of the patient. Over half of the participants suggested that the potential cause of exacerbations was influenced by environmental factors, including time of year and corresponding weather.

*‘..so whenever you get a change in weather from cold to hot, hot to cold, and things like that, it tends to be a bit of an uptake.’ (32110)*

*‘I think there’s an environmental factor, depending on what’s set this exacerbation off and whether we’ve been able to remove them from that.’ (4729)*

*‘If it is initiated by an allergen then that takes time to resolve as the body is fighting on two fronts. The same with an underlying condition, the body will already be weakened so it takes longer to resolve multiple issues with multiple systems and the weaker you are the less you have to fight with.’ (3215)*

Additionally, the majority of participants said that patient age should be considered, but agreed that whilst it may be a compounding variable in respiratory disease, it is not a cause in itself. It is believed that older respiratory patients often have comorbidities that may require a change in overall management.

*‘The older they get the more complications there are, like COPD.’ (1617*)

### Adaptation of patient management approaches

All participants reported compliance with JRCALC guidelines when administering salbutamol. However, variations in approach were demonstrated by many when it came to the administration of supporting drugs including ipratropium bromide and hydrocortisone.

*‘I don’t tend to step away from the algorithm as it is pretty straight forward. Salbutamol to start, then ipratropium bromide as a follow up with hydrocortisone or adrenaline as required.’ (32212)*

*‘If we’re seeing like a response from the salbutamol and they’re getting better, then we would stick with that.’ (1616)*

Considering the patient’s condition and subsequent response to initial treatment, some participants expressed quite reactionary attitudes. Of note is the use of hydrocortisone, with participants reporting only administering when nothing else seemed to be effective, resulting in infrequent use.

*‘I’ll use hydrocortisone after two nebs if the patient is still really struggling and it’s not having much effect and they are haemodynamically compromised, and still can’t speak in full sentence.’ (16111)*

In contrast some participants are more regimented with their approach, suggesting that its use is more commonly part of their practice.

*‘As soon as the patient hits the criteria of severe, but I know from my practice people are always really surprised when I do get it out and I think that works amazing.’ (1618)*

Based on their experience, all participants agreed that patients with asthma often have complex medical conditions which primarily involve cardiorespiratory or immunoglobulin E (IgE) sensitization pathways (e.g. allergies).

The most common compounding condition noted was Chronic Obstructive Pulmonary Disease (COPD), with the majority of participants stating its presence within the elderly population.

*‘I wouldn’t like to say that COPD is a comorbidity of asthma. It’s basically a mixture of conditions.’ (3215)*

*‘I suppose quite frequently you get the patients who potentially could be presenting as a COPD patient or have a history of COPD and they say asthma, and you have to kind of figure out’ (1619)*

When a patient has both asthma and COPD, most participants emphasised the need to adjust treatment to take both into account. The process of delivering oxygen for 6 min at a time is often combined with nebulisation to prevent hypercapnic respiratory failure.

*‘We change treatment around a central guideline, so COPD patients may receive the same treatment, but the timings may be changed to accommodate the six minutes.’ (1646)*

The potential for mimics and confounding presentations was further elaborated by some, noting that misidentification and misdiagnosis can often occur in the absence of a complete medical history, or confirmatory test results. This may initially lead to them treating patients more generally.

*‘Is it COPD? Is it asthma? Is it Congestive Heart Failure?’ (32212)*

Participants expressed similar confusion when attending patients experiencing some form of IgE pathway reaction. Despite the precedented link between asthma and allergies, just over half of the participants said it was a factor they were aware of, or had experienced.

*‘When looking at the lower age ranges you often get patients who have allergic conditions which could act as the trigger for their asthma in the first place…’ (3215)*

*‘I recently had a patient that was asthmatic and was having either anaphylaxis or just an allergic reaction that set the asthma off, but it was quite severe in terms of stridor which I think was due to the allergic reaction rather than the asthma, so instead of going down the asthma protocol, went down the anaphylaxis protocol.’ (1626)*

The majority of participants focused on changes in overall treatment due to additional respiratory or cardiac conditions, while more than half also mentioned the potential for secondary conditions and common risk factors to affect the stability of the patient’s asthma, how it presents and the treatment thereof.

*‘I’m just trying to remember the last chap I saw he was quite poorly. I think he was diabetic Type 2 and I think he had high blood pressure as well.’ (1616)*

*‘There are those patients that have asthma and other related conditions such as obesity, where one supports the other to some extent, which then could lead to additional conditions such as diabetes or cardiac conditions.’ (32212)*

*‘Even pregnant patients, stroke patients, diabetic patients, cardiac patients and all the others have their own requirements, so it is more about adding to the treatment.’ (1646)*

Alongside the physiological changes, some participants noted the importance of recognising psychological changes and their impact on overall management. Participants discussed variables surrounding comprehension, language and a range of mental health conditions, noting that the changes needed to treat a particular patient may result in suboptimal treatment for the exacerbation at that time.

*‘The only other things are the patients with learning difficulties or dementia, which is more of a challenge as you need to get them to understand what you are doing and strategies to get them to do what you need them to do, instead of something impacting your treatment strategy.’ (4729)*

Overall, participants expressed a dynamic approach to treating patients with asthma. Most suggested prioritising airway treatment, but stated that the focus of treatment could change depending on other observations.

*‘We tend to supplement how we treat patients so I may nebulise someone whilst assessing their heart and then I may treat that as well. It is more about stacking treatments.’ (32212)*

*‘In general it depends on how well controlled they are. If they don’t get much better then there might be something else happening. It isn’t always clear, and I wouldn’t necessarily know……. You change your treatment if needed as your proximity to hospital may be longer and modifying your treatment to manage that presentation, as opposed to that presenting condition.’ (1618)*

*‘There’s also an element of anxiety that causes problems. Are they having effects from the drug or from the way they are?’ (1618)*

Of the fifteen participants, eight reported that they had not knowingly treated a patient with a complaint the patient was unaware of, although the majority of participants highlighted that it is sometimes difficult to distinguish between what is a comorbidity and what are negative side effects from the treatment.

### Severity of side effects

All participants reported that the most commonly observed side effect when administering nebulised salbutamol was cardiac abnormalities. Participants further confirmed that an overall increase in heart rate is common, which may lead to ultimate tachycardia.

*‘I’d say that the most obvious ones that I would find are measurable changes in heart rate and blood pressure from the B*_*2*_ *stimulation. I would say with all adults that I am continuously monitoring, I’d be surprised if there wasn’t an increase in heart rate and usually blood pressure as well.’ (16211)*

These cardiac changes do not always occur in isolation, with most participants stating that muscle tremors, although not present in every patient, are commonly witnessed.

*‘Like I say, I’d see tremors in one in every five patients I give salbutamol to… The same with tachycardia, I see that maybe every two or three.’ (16111)*

*‘I would say about fifty percent of the time the patient ends up with like tremors or shaking.’ (4736)*

Although increased heart rate was the main side effect, participants did not necessarily see it as a potential problem. Half of the participants would only raise concern when rates reach levels of supraventricular tachycardia (a rate of >150 bpm). To clarify this, several participants said that these effects mainly depend on the patients themselves, taking into account individual characteristics and the different presentations witnessed.

*‘I think if the patient became symptomatic and if the patient decompensates with it, then I would probably stop, but I suppose I look at ABC’s and if I’m still significantly concerned about the wheezing and stuff.’ (1619)*

*‘If their heart rate gets too high the risk is that they may have cardiac issues as well, so it’s a balancing act.’ (1617)*

*‘Very much I think it’s down to the individual patient. Obviously, they need to breathe. Am I getting enough air in or are they getting enough air in, or am I causing another problem with the gaseous exchange and stuff due to the heart rate…’ (4729)*

Participants did not identify other side effects that could potentially be problematic for patients and/or the care provided. Instead, they were considered incidental and included as signs, symptoms, or effects of treatment.

*‘You get a lot of headaches reported and sometimes they feel the odd palpitation, but we only hear about that if the patient recognises what is happening and the obvious dry mouth as well, but that is more from the nebuliser effect opposed to the drug itself.’ (3215)*

*‘I’ve had a few people complaining about headaches after the nebuliser, but I’m not sure if that’s just the extra effort of breathing and the fact that they are having an asthma attack and the stress, or whether it’s just the nebuliser….’ (1626)*

## Discussion

The findings from this study demonstrate an approach to practice that is in line with the Clinical Reasoning Cycle^19^, where paramedics instinctually collect information about their patients and use both existing guidelines, patient demographics and their own clinical experience to inform their clinical decision-making. The Clinical Reasoning Cycle^19^ illustrates the cognitive processes of acquiring and processing information, from perception to reasoning and demonstrates how medical professionals can effectively treat patients with various contradictory or worsening conditions. Ritz et al. ^26^ discuss this challenge, suggesting that healthcare is not an exact science, and it is not always possible to label patients by their presenting medical condition^19^.

The analysis found that paramedics effectively followed the guidelines while responding to changes in the patient’s condition and relevant medical history. Participants highlighted this when referring to the older population where the presence of asthma is often complicated by other respiratory conditions such as COPD, or other compounding factors, such as those caused by a cardiac history. Additionally, participants reported that patients often experience side effects such as arrhythmias, tremors and headaches, but these are often accepted as part of the treatment process and only considered problematic when levels are reached that overshadow the presenting asthma exacerbation.

Similarly, the influence of psychological factors can also directly affect the current condition and cause its deterioration. When treating exacerbations, paramedics try to address anxiety and prevent it from becoming a contributing factor. This is discussed by Stubbs et al. ^27^ who note the impact that anxiety and associated depression can have on the control of asthma, often resulting in poorer health outcomes. These views and subsequent approaches demonstrate a more holistic understanding of the presenting complaint where clinicians look at common linking and underlying factors that may be present. This was reinforced with reference to diabetes as a significant comorbidity, strengthened with discussion of shared risk factors such as weight control and mobility, something Stubbs et al. ^27^ also note and which is commonly discussed within literature relating to the two conditions^28^. The way paramedics treat patients is based on the clear understanding that no two patients are the same, and depending on the patient’s current situation, paramedics will objectively decide on a treatment plan that may involve an adaptive application of guidelines. Despite three clinical grades of paramedics participating in this study, their roles did not appear to influence their perception or decision-making when managing asthma patients. The reason may be as one participant stated, because ‘*the algorithm ….. is pretty straightforward,’* meaning they simply follow this until treatment needs to be escalated or deescalated, or when contributory factors such as comorbidity require attention.

Despite the lack of homogeneity in asthma patients, the priority in patient management is to ensure airway patency before providing further treatment. Although all participants in this study shared this view, their deeper understanding that the respiratory system does not function independently was apparent and supported by Sarkar et al. ^10^ who discussed the importance of homoeostasis in maintaining a suitable V/Q balance. Participants often referenced the possibility that cardiac changes could affect the patient’s breathing and corresponding mechanisms. Further consideration showed that these effects may be due to other medical conditions or drug side effects. However, it is unclear at what level these changes become problematic.

Consistent with current literature^7,8^ and clinical expectations, a general acceptance that cardiac-related side effects may occur when managing asthma patients with β₂ agonists is shown. However, in this instance despite a clinical reasoning approach, clinicians may overlook them as a potential problem, which would not be the case if the effects presented outside of this specific treatment. The reflective component of the Clinical Reasoning Cycle^24^ can help conceptualize this, as each asthma patient cared for is exposed to a greater variety of systemic changes, thereby increasing understanding and expectations for the next patient.

Knowing that a patient has a comorbidity which may impact the presenting condition is important. However, surreptitiously replicating those same effects through the use of treatment brings an added risk^7–10^. The participants demonstrated an awareness of contributing factors present when asthma patients experience an exacerbation of their condition, a point considered by Boulet^29^ who outlines that underlying conditions can impact the severity of asthma in several ways, from changing the phenotype to altering presentation.

Patient-centred care heralds the view that patients are individuals, a concept Entwistle and Watt^30^ supported when discussing communication and patient empowerment, suggesting that clinicians should also be mindful not to manage patients according to a set list of expectations. Chouchane et al. ^31^ further advocate this view when citing human factors as key to effectively managing patients, explaining that sometimes the most effective drug may not be suitable for all patients.

Participants clearly demonstrated that they consider the complexity of patient’s individual needs and individual treatment needs, especially when conditions such as stroke, pregnancy, diabetes, and even anxiety are identified. This level of awareness suggests that paramedics and other clinicians may consider varied physiological, psychological and behavioural changes, but as Rosendal^32^ Pizzorno^33^ and Richens et al. ^34^ suggest, if we are only treating the presence of symptoms without identifying the causes, these may present themselves as larger problems in the patient’s future. An example drawn from these data is: If administration of salbutamol causes reciprocal changes, including an increase in blood glucose levels, how do clinicians know if their patient is also hyperglycaemic at the time of treatment if they do not routinely assess Capillary Blood Glucose levels for asthma patients^35^.

The findings suggest that the level of understanding demonstrated by all paramedics regarding causation, treatment and compounding factors shows a preference to manage their patients on a ladder of escalation, as opposed to a scripted ‘one-size-fits-all’ approach. The data suggests varying degrees of acceptance that other medical conditions may be present, or that treatment may result in physiological changes that affect the patient’s health status. However, a pre-emptive approach to compensate for this is not always demonstrated. Future research could investigate the specific comorbidities and/or side effects experienced by asthma patients and ascertain the point at which clinicians perceive non-therapeutic effects to be clinically significant for patients, both in the short and long term.

The study recruited at a time when the UK was starting to recover from the COVID-19 pandemic, meaning there were still a number of effects impacting how patients were managed. This was both from the patients’ and clinicians’ perspective and included not only the clinical complications associated with COVID-19, but also a reduction in calls to milder and more moderate asthma exacerbations^36,37^. Paramedics may have seen fewer asthma patients during this time compared to the previous two years, and when they did their considerations with these patients would have been complicated by consideration of the COVID-19 infection.

The COVID-19 pandemic further meant we undertook all interviews online. Although this approach may have had benefits, we accept that in-person interviews are preferred to build a rapport between interviewers and interviewees. However, this is not thought to have significantly impacted the data collected for this study.

Although 15 interviews may seem like a small number on the surface, the literature suggests that the number of participants in such studies should not be considered in isolation, but rather as part of the larger element of data adequacy which looks at the quality, richness and level of commonality or differences between data^20,21^. We therefore believe that the data collected and presented is suitably representative of the views/actions of paramedics across the participating Trust.

In conclusion, asthma is an inflammatory disease with diverse phenotype characteristics. Recognising the factors present during an exacerbation allows paramedics to respond effectively to individual complexities through consideration of changing phenotypes, compounding comorbidities and response to pharmacological treatment. Consideration of the various physiological (e.g. blood glucose level), psychological (e.g. anxiety) and behavioural changes (e.g. sedentary time) experienced by patients allows paramedics to improve their level of clinical reasoning and dynamically adapt their management to provide optimised care in the short-term and potentially reduce any long-term health implications. These underlying considerations are often based on experience and developmental reflection, influencing drug selection and interpretation of the relevance/impact of any observed side effects or comorbidities.

## Supplementary information

Supplementary Information

Reporting Summary

## Data Availability

The datasets used and/or analysed during the current study are available from the corresponding author on reasonable request.
