# Supplementary material for: Clinical reasoning amongst paramedics using nebulised β₂ agonists to treat acute asthma exacerbations: a qualitative study
Source: NPJ Prim Care Respir Med. 2024 Sep 6;34:24. doi: 10.1038/s41533-024-00383-w (PMC11379812; doi:10.1038/s41533-024-00383-w)
Supplement: Supplementary file 1 — Supplementary Information [file 41533_2024_383_MOESM1_ESM.pdf]

Supplementary Table 1. Interview Schedule

A copy of the semi-structured interview schedule used by the interviewer.

**Prevalence/Background Experience**<br>*'The national figures say that over 5 million people suffer with asthma and seek support from the range of healthcare providers, so how often would you say you attend an asthma patient within your current role?*<br><br>1. What level of severity are they seeing?<br>2. What is the general age range they attend?
**Standard Management**<br>*'Tell me about how you would usually manage a patient presenting with an acute exacerbation of asthma?'*<br><br>1. What would be their main drugs of choice when managing an exacerbation of asthma? *(For the purpose of this study we are looking at those drugs that are nebulised).*<br>2. With their first line intervention being Salbutamol, in their experience how many doses would they say are commonly needed to get an exacerbation under control?<br>3. Do they always use Ipratropium Bromide when managing asthma patients? What guides their decision?<br>4. How often do they use hydrocortisone to supplement their treatment?
**Pharmacodynamics**<br>*'The therapeutic effects of Salbutamol are well known so for this study, we would instead like to focus on its side effects. Tell me about your experiences of side effects presenting following the use of nebulised Salbutamol in practice.'*<br><br>1. Have they seen any of the suggested side effects of Salbutamol manifest in practice? (JRCALC: Tremor, Tachycardia, Palpitations, Headache, Tension, Vasodilation, Cramps, Rash) Are they seen regularly? Which are most frequent? Any other side effects seen?<br>2. Have they had to discontinue administering Salbutamol due to any of the stated side effects?<br>3. At what point do they believe that these side effects become problematic to the patient?<br>4. How do they balance the risks of any side effects against the treatment being provided
**Comorbidity and Multimorbidity**<br>*'As we know many patients have multiple conditions and this may or may not impact how we manage that patient. Do you have any examples of when this may have been the case with an asthma patient?'*<br><br>1. Have they treated asthma patients that have a significant comorbidity and if so what?<br>2. Have they considered changing their treatment due to the comorbidity and if so why?<br>3. Any experience of patients with no known comorbidity seeming to present with another condition whilst managing their asthma? If so do they know why this occurred?
**Timeframe**<br>*'Asthma as with any condition has a timeline and a series of points within that timeline such as onset of symptoms, therapy initialised, and exacerbation resolved. What is your experience of the time taken to control an exacerbation of asthma?'*<br><br>1. Why do they think exacerbations take this length of time to abate?<br>2. Have they seen a biphasic response occur in an asthma patient they are treating? Do they know why this happened?

Supplementary Table 2. Sample Role Descriptors

An explanation of the interview participants' clinical roles.

**Experienced Paramedic**	Qualified through a nationally accepted education programme and holds a minimum of two years post-registration (HCPC) experience. Primarily the lead clinician with experience and knowledge base to undertake autonomous care across a range of urgent/emergency situations, within a range of settings.
**Newly Qualified Paramedic (NQP)**	Qualified through a nationally accepted education programme and holds less than two years post-registration (HCPC) experience. During this time, a Clinical Preceptorship Programme is undertaken to support the consolidation of skills gained as a student paramedic.
**Paramedic Practitioner (PP)**	An experienced HCPC registered Paramedic who has completed an accredited university pathway to practice at an advanced clinical level, allowing them to manage an extended range of urgent clinical conditions.
